# Sustained Release of αO-Conotoxin GeXIVA[1,2] via Hydrogel Microneedle Patch for Chronic Neuropathic Pain Management

**DOI:** 10.3390/md23040161

**Published:** 2025-04-07

**Authors:** Rongyan He, Mingjuan Li, Weitao Li, Wenqi Li, Shuting Xiao, Qiuyu Cao, Huanbai Wang, Dongting Zhangsun, Sulan Luo

**Affiliations:** 1 Guangxi Key Laboratory of Special Biomedicine, School of Medicine, Guangxi University, Nanning 530004, China; herongyan@gxu.edu.cn (R.H.); autumnrain1999@163.com (Q.C.); zhangsundt@163.com (D.Z.); 2 Key Laboratory of Tropical Biological Resources of Ministry of Education, Hainan University, Haikou 570228, China; hbwang93@163.com

**Keywords:** αO-conotoxin GeXIVA[1,2], neuropathic pain, sustained release, microneedle patch, hydrogel encapsulation

## Abstract

Chronic neuropathic pain severely impairs quality of life, with current therapies often causing adverse effects. Our research group identified αO-conotoxin GeXIVA[1,2] as a potent analgesic candidate derived from marine cone snails. However, its clinical application is limited by rapid clearance and complex administration. This study developed a sustained-release hydrogel microneedle patch encapsulating GeXIVA[1,2] to address these challenges. Optimized 4:3 (*w*/*w*) polyvinyl alcohol (PVA)–sucrose hydrogel formulation achieved 98.6% structural integrity and controlled swelling (ratio = 1.9 at 48 h). The microneedles demonstrated uniform conical morphology (height: 889 ± 49 µm, base: 381 ± 26 µm) enabling epidermal penetration. In spared nerve injury (SNI) models, a single microneedle patch application increased mechanical paw withdrawal thresholds from 0.056 g to 0.7269 g, maintaining efficacy for 3 days. Chronic constriction injury (CCI) models showed comparable pain relief. Notably, microneedle patch treatment improved locomotor function in SNI mice (total movement: 1518 cm vs. 1126 cm untreated). This hydrogel microneedle patch platform extends GeXIVA[1,2]’s analgesic duration from hours to days through sustained release, while resolving administration challenges through transdermal delivery, expanding the potential applications of GeXIVA[1,2], and demonstrating a promising strategy for the chronic neuropathic pain management.

## 1. Introduction

Chronic neuropathic pain, resulting from injury or disease affecting the peripheral or central nervous system, is an incurable and debilitating condition characterized by intense pain [1,2]. This relentless suffering exhausts patients physically and mentally, significantly diminishing their overall quality of life [3]. The prevalence of chronic neuropathic pain is rising annually, affecting 3.3–8.2% of the general population. Notably, the condition is increasingly occurring among younger individuals, placing a growing and substantial burden on both society and the economy [4]. Chronic neuropathic pain is characterized by its prolonged duration, typically lasting for over three months to years; more severely, some patients endure pain for more than a decade or even throughout their entire lives [5]. The clinically therapeutic approaches for chronic neuropathic pain encompass medication, neuromodulation, and neurological intervention techniques (e.g., electrical nerve stimulation), and surgical treatments [6]. Neuromodulation and neurological interventions are effective, but their high costs and reliance on specialized medical facilities pose significant challenges for long-term treatment. Medication remains the fundamental and essential strategy [7]. For instance, non-opioid drugs (e.g., non-steroidal anti-inflammatory drugs and α-adrenergic receptor agonists) and weak opioid analgesics (e.g., tramadol and codeine) are typically used for mild to moderate pain. However, these medications can induce adverse effects such as nausea, vomiting, constipation, and urinary retention [8]. Moreover, they are often ineffective for patients suffering from severe pain. Opioid analgesics (e.g., morphine, oxycodone, and fentanyl) can be used to alleviate severe pain. Nevertheless, the risks of addiction, tolerance, and severe dose-limiting toxicity associated with opioids hinder their suitability for long-term daily use [9]. Consequently, there remains a critical unmet need for the development of sustainable, long-term strategies to effectively manage chronic neuropathic pain.

Conotoxins are peptide neurotoxins secreted by marine mollusks, known for their ability to specifically target ion channels (e.g., Na^+^, K^+^, and Ca^2+^ channels) or receptors (e.g., nicotinic acetylcholine receptor (nAChR) and norepinephrine receptors) [10]. Our research group identified GeXIVA[1,2] from the *Conus generalis*. GeXIVA[1,2], a peptide composed of 28 amino acids (sequence: TCRSSGRYCRSPYDRRRRYCRRITDACV) containing four cysteine residues forming two disulfide bonds (Cys2-Cys9 and Cys20-Cys27), selectively targets the α9α10 nAChR, which is a target of analgesic therapies, with potent biological activity (IC_50_ = 4.6 nM) [11]. The arginine-enriched composition of GeXIVA[1,2] not only mediates receptor binding through electrostatic interactions, which is critical for its bioactivity, but also confers good aqueous solubility due to the high content of these positively charged residues [12]. It has demonstrated remarkable analgesic efficacy in various animal models, including mice, rats, and beagles [13,14,15] with its activity exceeding that of morphine by approximately 1000-fold and surpassing gabapentin by more than 2000-fold, based on molar mass comparisons in chronic neuropathic pain models [11]. Moreover, our group has proved that GeXIVA[1,2] is biologically safe without drug resistance or addictive properties [16]. These unique properties make GeXIVA[1,2] a promising candidate for the management of chronic neuropathic pain.

However, GeXIVA[1,2] faces a significant challenge in the complexity of the administration route for its nature of peptides. Conotoxins are typically administered via intracranial, intrathecal, or intramuscular injection [17,18], which poses potential risks and requires professional medical operators, thereby bringing substantial time costs and financial burdens. Moreover, needle phobia is a common issue, affecting approximately 20% of adults and 50% of children [19]. For these individuals, the fear of needles often makes injection-based therapies difficult to accept, thereby limiting the applicability of conotoxin peptides in this patient population. Currently, there are no studies focused on improving the administration route for GeXIVA[1,2]. Therefore, the development of a more convenient and patient-friendly delivery method for GeXIVA[1,2] is of great importance.

Hydrogel microneedle patches provide an efficient and self-administrable method for the localized delivery of conotoxin GeXIVA[1,2], making them an ideal platform for managing chronic neuropathic pain [20,21]. Microneedle patches consist of arrays of micron-sized needles, typically 25–1000 μm in length and 1–100 μm in tip diameter [22,23]. They painlessly penetrate the stratum corneum, creating microchannels for transdermal drug delivery [24]. This method offers advantages, including minimal invasiveness, pain-free application, and the convenience of self-administration. On the other hand, hydrogels are highly hydrophilic polymers with a three-dimensional network structure [25]. Owing to their excellent biocompatibility and superior drug-loading capacity, hydrogels have found wide applications in the field of drug delivery [26]. By encapsulating GeXIVA[1,2] within hydrogels, the peptide is shielded from the plasma environment, as the hydrogel acts as a protective barrier against proteases that prevents protease-mediated cleavage and reduces the degradation rate of GeXIVA[1,2]. Furthermore, the hydrogel microneedle patches enable the formation of a sustained-release system, where GeXIVA[1,2] is gradually released through the swelling and slow degradation of hydrogels under the skin. The drug concentration can be controlled by modulating the hydrogel’s swelling ratio and degradation rate in vivo.

**Figure 1:**
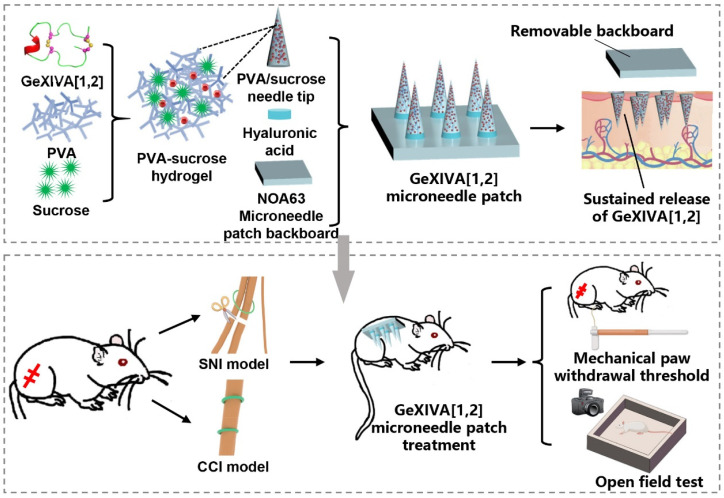
Schematic of the sustained-release αO-conotoxin GeXIVA[1,2] hydrogel microneedle patch for chronic neuropathic pain management. Preparation of GeXIVA[1,2] hydrogel microneedle patch; treatment of spared nerve injury (SNI) and chronic constriction injury (CCI) mouse models with GeXIVA[1,2] hydrogel microneedle patch.

In this study, we designed a sustained-release hydrogel microneedle patch loaded with αO-conotoxin GeXIVA[1,2] for the management of neuropathic pain (Figure 1). The microneedle tips were fabricated using a polyvinyl alcohol (PVA)–sucrose hydrogel to encapsulate GeXIVA[1,2], while Norland Optical Adhesive 63 (NOA63) served as the rigid backboard to provide mechanical support for skin penetration. To achieve rapid application and sustained release, we designed a rapid-separation microneedle patch that enables rapid separation of the microneedle tips from the backboard by incorporating a fast-dissolving hyaluronic acid (HA) layer as an adhesive interface. This innovative hydrogel microneedle patch prolongs the therapeutic duration of GeXIVA[1,2], reduces the frequency of administration, and provides a patient-friendly, self-administrable delivery method. This study not only expands the potential applications of GeXIVA[1,2], but also introduces a promising new strategy for the effective management of chronic neuropathic pain.

## 2. Results

### 2.1. Optimization of Composition Ratios of Hydrogel Microneedle Patches

The composition ratios of hydrogel microneedle patches play a crucial role in determining their overall performance, particularly in terms of mechanical strength, drug-loading capacity, sustained-release properties, and structural integrity. PVA has emerged as a promising material for fabricating microneedle patches for peptide drug delivery, owing to its excellent biocompatibility, tunable porosity, and unique phase transition characteristics [27]. In its dry state, the material exhibits glass-like rigidity, enabling effective penetration of the epidermal layer, while upon absorption of skin interstitial fluid, it undergoes a phase transition to a soft hydrogel state, facilitating controlled drug release while maintaining structural integrity and minimizing potential damage to subcutaneous tissues [28]. Owing to its multiple hydroxyl groups, sucrose can form reversible hydrogen bonds with PVA when incorporated into PVA hydrogels [29]. These intermolecular interactions induce molecular-level crosslinking between PVA chains, resulting in the formation of a stable PVA–sucrose hydrogel network.

**Figure 2:**
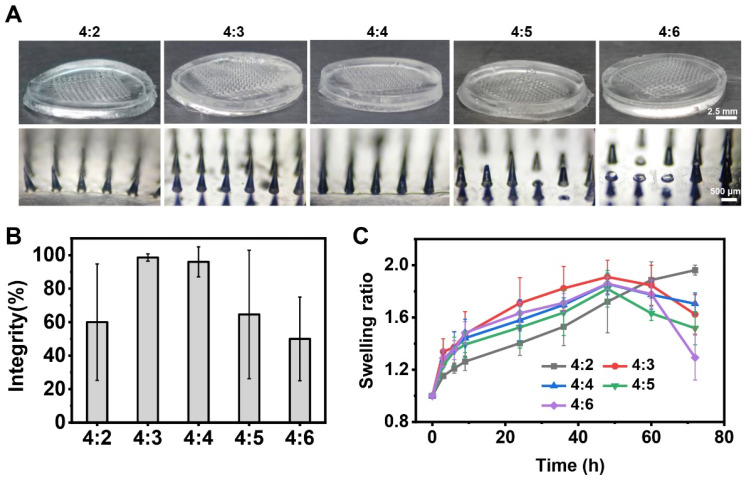
Optimization of material ratios for hydrogel microneedle patches. (**A**) Overall photographs and localized microscope images of microneedle patches with varying PVA and sucrose ratios. (**B**) Integrity of microneedle patches with different PVA–sucrose compositions. (**C**) Swelling ratios of microneedle patches.

To develop microneedle patches with optimal properties for GeXIVA[1,2] delivery, we systematically optimized the PVA–sucrose hydrogel composition. Four different weight ratios of PVA to sucrose (4:2, 4:3, 4:5, and 4:6 *w*/*w*) were investigated to fabricate the microneedle patches. The microneedles exhibited intact conical shapes without defects, fractures, or deformations, and no obvious voids or imperfections were observed within the microneedles, which were considered structurally intact. The microneedle patches prepared with PVA:sucrose ratios of 4:3 and 4:4 demonstrated high structural integrity, with integrity rates of 98.6% (SD = 2.19%) and 96.0% (SD = 8.94%), respectively (Figure 2B). These results indicate that these PVA–sucrose formulations enable reliable fabrication of structurally intact microneedle patches. In contrast, microneedle patches fabricated with other PVA:sucrose ratios exhibited lower structural integrity. Patches fabricated with a PVA:sucrose ratio of 4:2 exhibited mechanical fragility, leading to frequent fracture during the demolding process and consequent tip retention within the mold cavity. The increased viscosity of solutions with PVA:sucrose ratios of 4:5 and 4:6 hindered complete infiltration into the micropores of the polydimethylsiloxane (PDMS) mold, ultimately leading to reduced microneedle integrity.

Given the close relationship between the swelling properties of microneedle materials and drug release kinetics, we measured the swelling ratios of microneedle formulations with varying PVA–sucrose hydrogel compositions through implanting them at the dermal–muscle interface in an ex vivo model. From Figure 2C, we observed that hydrogel swelling behavior was time-dependent, with all hydrogel showing initial fluid absorption from the skin interstitial space. The PVA:sucrose = 4:2 hydrogel demonstrated a consistent increase in swelling ratio over time, whereas hydrogels with other compositions exhibited a distinct swelling-degradation profile, characterized by subsequent mass reduction. Among them, the 4:3 hydrogel exhibited optimal swelling performance within the first 48 h, achieving a swelling rate of 1.9. At the same time, the microneedle patch fabricated using this ratio demonstrated the highest integrity rate. Therefore, we selected the PVA:sucrose = 4:3 hydrogel as the substrate material for the microneedle patches in subsequent experiments.

### 2.2. Fabrication and Characterization of Hydrogel Microneedle Patches

**Figure 3:**
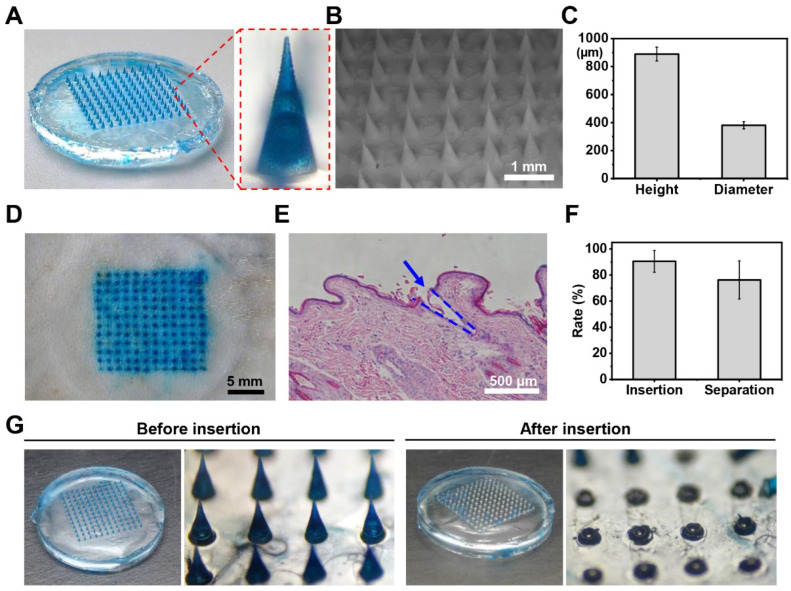
Characterization of GeXIVA[1,2] hydrogel microneedle patches. (**A**,**B**) Overall photographs and localized scanning electron microscope (SEM) images of GeXIVA[1,2] microneedle patches. (**C**) Height and diameter measurements of microneedles. (**D**) Mouse back skin appearance after insertion and removal of dye-loaded microneedle patches. (**E**) H&E-stained skin tissue sections after microneedle patch insertion. (**F**) Insertion and separation rates of microneedle patches. (**G**) Images of microneedle patches before insertion into mouse skin and immediately after removal from mouse skin following a 1 min retention period.

We fabricated microneedle patches loaded with methylene blue instead of GeXIVA[1,2] to observe the morphology of microneedles (Figure 3). From Figure 3A, we can see that the microneedle patch displays a well-organized 10 × 10 array of uniformly arranged conical microneedles. Figure 3B further demonstrates the concentration of methylene blue within the microneedle structure. We measured the dimensions of 300 microneedles from six patches, obtaining an average height of 889.05 ± 49.35 µm and a root diameter of 381.02 ± 26.19 µm (Figure 3C). Since human skin comprises the epidermis (76.9–267.4 µm thick) and the dermis (2115–5888 µm thick) [30], the microneedles are designed to penetrate the epidermis and deliver drugs into the upper dermis. This design avoids damage to blood vessels and nerves located in the deeper dermis, thereby preventing bleeding and pain. To evaluate the transdermal drug delivery capability of the microneedle patches, we applied the methylene blue-loaded patch to isolated skin. We observed a well-defined array of microholes on the skin surface and successful retention of methylene blue within the skin tissue, confirming effective transdermal drug delivery (Figure 3D). Figure 3E presents a hematoxylin–eosin (H&E)-stained skin section image, clearly demonstrating that the microneedle patches successfully penetrate the epidermis and create microchannels in the dermis.

Conventional microneedle patches deliver drugs through complete dissolution of the microneedles in the skin’s interstitial fluid, enabling subcutaneous drug release. However, this mechanism requires prolonged patch wear time, potentially increasing patient risks such as infection and allergic reactions. To address this limitation, we developed a novel rapidly detachable microneedle patch featuring a unique three-layer structure: drug-loaded PVA–sucrose tips, a rigid backboard, and a fast-dissolving hyaluronic acid connecting layer. Upon skin insertion, the connecting layer rapidly dissolves, enabling immediate separation of the tips from the backboard. This design allows the microneedle tips to remain embedded in the skin for sustained drug release while permitting prompt removal of the backboard, significantly reducing patch wear time. To evaluate the separation capability of the microneedle patches, we conducted ex vivo skin experiments and quantified both the skin penetration efficiency and tip-backboard separation rate (Figure 3F,G). The results demonstrated that approximately 90% of the microneedles successfully penetrated the epidermal layer, with 76% of the tips remaining in the dermis after backboard removal (Figure 3F). Figure 3G presents comparative images of the microneedle array before and after skin insertion, clearly showing the intact array prior to application and successful tip separation following insertion (Supplementary Video S1).

### 2.3. In Vitro Release of the GeXIVA[1,2] Microneedle Patches

**Figure 4:**
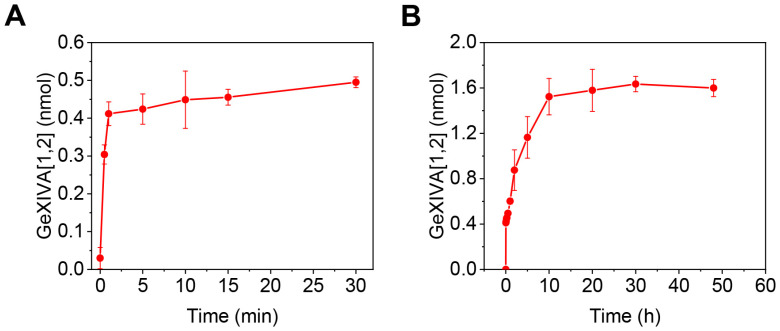
Cumulative in vitro release of GeXIVA[1,2] from hydrogel microneedle patches. (**A**) In vitro release of GeXIVA[1,2] from hydrogel microneedle patches over 30 min; (**B**) The sustained-release profile of GeXIVA[1,2] from hydrogel microneedle patches within 48 h.

To investigate the release profile of GeXIVA[1,2] from the microneedle patches, we synthesized high-purity GeXIVA conotoxin peptide. The measured mass was 3451.6 Da, which are in good agreement with the expected value (3451.96 Da) (Figure S1). We loaded it into hydrogel microneedle patches, forming GeXIVA[1,2] hydrogel microneedle patches. We conducted in vitro release studies through placing them into 10 mL phosphate buffer saline (PBS, pH = 7.4) at 37 °C, with quantification by reverse-phase high-performance liquid chromatography (RP-HPLC). As shown in Figure 4A, the patches exhibited an initial burst release, delivering 0.41 nmol (20.5% of total loading) within the first minute, followed by a gradual increase over the subsequent 30 min. This rapid initial release probably results from surface-adsorbed GeXIVA[1,2] that dissolves upon contact with the solution. Subsequently, hydrogel swelling facilitates sustained release through the three-dimensional network pores, leading to a slower release rate after the initial burst. Figure 4B demonstrates the sustained-release profile, revealing accumulation to 1.52 nmol (76% of total loading) within the first 10 h, followed by a gradual release within 30 h. We also added blue dye to the microneedles to visualize the in vitro drug release process (Figure S2). The sustained-release mechanism depends on hydrogel swelling, which in vivo would be driven by absorption of skin interstitial fluid. Considering the lower volume of skin interstitial fluid in vivo than in vitro release testing, we anticipate that the in vivo release of GeXIVA[1,2] would exhibit an extended duration.

### 2.4. Evaluation of Drug Release In Vivo

**Figure 5:**
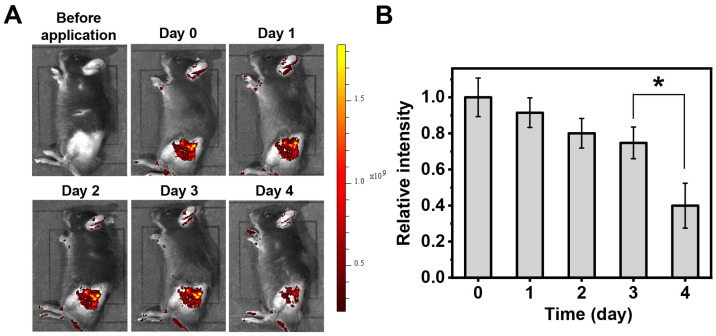
Cumulative in vivo release of model drugs over 4 days. (**A**) Fluorescent images before applying microneedle patches, and after application (Day 0, Day 1, Day 2, Day 3, Day 4). (**B**) Fluorescence quantification of model drugs at each time point. (* *p* < 0.05).

To evaluate drug release in vivo, we utilized fluorescently labeled dextran-loaded microneedle patches, with dextran selected as a molecular weight analog of GeXIVA[1,2], to assess drug release in vivo by visualization of fluorescent tracers. Figure 5A shows fluorescence images of mice before and 0 to 4 days after applying microneedle patches, where we can see significant fluorescence in the mice’s depilated hind limbs after applying microneedle patches, and gradual signal attenuation over time. Quantitative analysis of fluorescence intensity demonstrates consistent signal maintenance during the first three days, without statistically significant daily variations (Figure 5B). However, a marked intensity reduction occurs between Day 3 and Day 4 (*p* < 0.05), indicating the concentration of drugs in the hind limb was considerably reduced on the fourth day. It suggests an initial phase of hydrogel swelling-mediated drug release during the first three days, followed by hydrogel degradation and subsequent drug metabolism and elimination on the fourth day.

### 2.5. GeXIVA[1,2] Microneedle Patches for Neuropathic Pain Management

**Figure 6:**
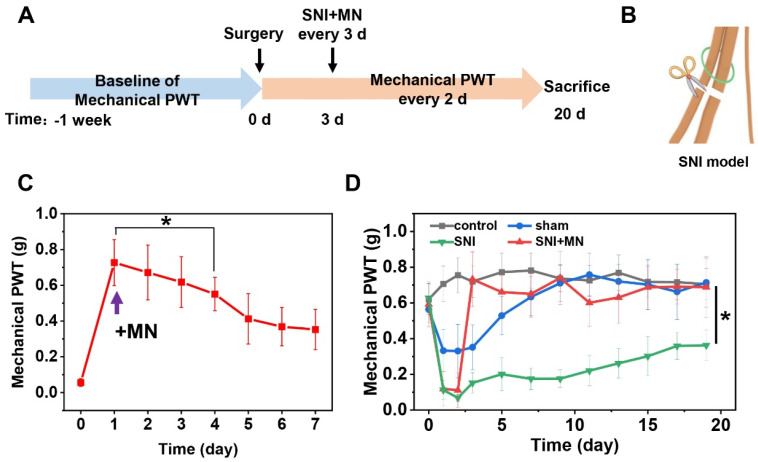
Analgesic effect of GeXIVA[1,2] microneedle patches in SNI neuropathic pain mice. (**A**,**B**) Timeline and surgical schematic of SNI experiments. (**C**) Mechanical PWT after single application of GeXIVA[1,2] microneedle patches (purple arrow indicates GeXIVA[1,2] microneedle patch application time). (**D**) Mechanical PWT in mice treated with GeXIVA[1,2] microneedle patches every three days (treatment initiated on Day 3). Groups: Control (healthy mice); Sham (sham surgery: skin and muscle incision without nerve damage); SNI (SNI model mice without treatment); SNI+MN (SNI model mice treated with GeXIVA[1,2] microneedle patches), * represents *p* < 0.05, n = 8.

To evaluate the therapeutic efficacy of GeXIVA[1,2] microneedle patches on neuropathic pain, we established both spared nerve injury (SNI) and chronic constriction injury (CCI) mice models, monitoring mechanical pain sensitivity through paw withdrawal threshold (PWT) measurements. Figure 6A,B illustrate the experimental timeline and surgical schematic for SNI model establishment. One week prior to surgery, we determined baseline PWT values and excluded mice exhibiting abnormal thresholds or impaired withdrawal reflexes. To assess the functional duration of a single GeXIVA[1,2] microneedle patch application, we administered the patch on postoperative 3 days (n = 8). We can observe a localized micropore array at the application site immediately after patch removal, which fully resolves within 50 min, restoring the skin to its original state, demonstrating the minimally invasive and rapid recovery characteristics of the microneedle patches (Figure S3). As shown in Figure 6C, the treatment significantly increased PWT from 0.056 ± 0.022 g (Day 0) to 0.7269 ± 0.1287 g (Day 1, *p* < 0.05). The therapeutic effect gradually declined over time, with PWT decreasing to 0.5507 ± 0.0934 g by Day 4 (statistically different from Day 1, *p* < 0.05) and further reducing to 0.3528 ± 0.1122 g on Day 7. These results demonstrate that a single GeXIVA[1,2] microneedle patch effectively alleviates SNI-induced neuropathic pain for approximately three days, maintaining mechanical pain sensitivity within the therapeutic range. Based on these results and evaluation of drug release in vivo, we selected a frequency of once every three days to apply GeXIVA[1,2] microneedle patches for subsequent in vivo pharmacological evaluations.

We conducted PWT measurement across four experimental groups: control, sham-operated (sham), SNI model (SNI), and SNI model treated with GeXIVA[1,2] microneedle patches (SNI+MN). As shown in Figure 6D, PWT measurements were performed daily for the first three postoperative days, followed by alternate-day assessments. All surgical groups exhibited significant PWT reduction within two days post surgery. This mechanical pain sensitization resulted from tissue damage in the sham group (0.3307 ± 0.1495 g) and was more severe in the SNI groups (0.0683 ± 0.019 g and 0.1113 ± 0.0960 g), confirming successful model establishment. Following GeXIVA[1,2] microneedle patch administration on Day 3, the SNI+MN group demonstrated significant pain relief, with PWT increasing to 0.7320 ± 0.1536 g, and no statistically significant difference was observed compared with the control group, showing statistically significant differences from the untreated SNI group (*p* < 0.05) throughout the treatment period. The analgesic effect of the GeXIVA[1,2] microneedle patch was nearly equivalent to that of direct intramuscular GeXIVA[1,2] injection observed on Day 1 (assessed at 2 h post administration) in our previous experiments [14], but the intramuscular injection exhibited no residual analgesic effect by Day 2. This result indicates that the GeXIVA[1,2] microneedle patches have a significant analgesic effect on the SNI model, and the analgesic effect persisted for at least three days.

**Figure 7:**
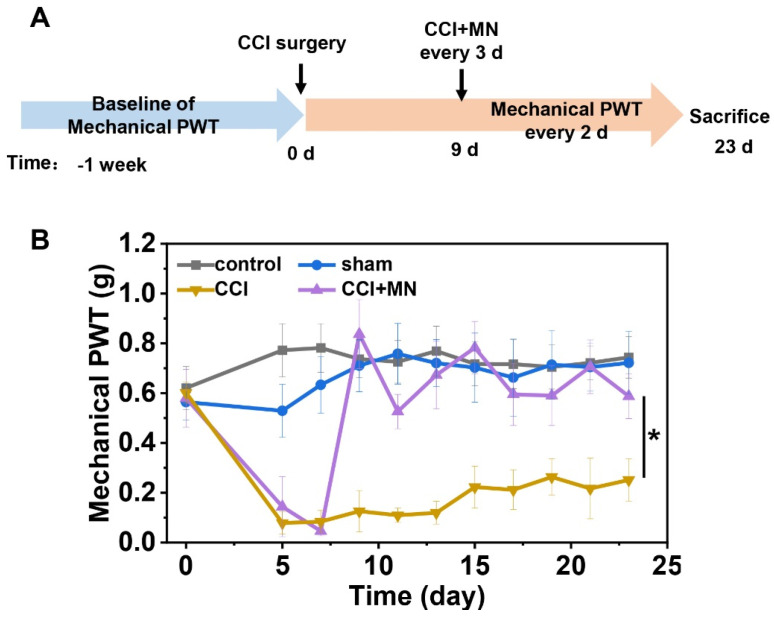
Analgesic effect of GeXIVA[1,2] microneedle patches in CCI neuropathic pain mice. (**A**) Timeline of CCI experiments. (**B**) Mechanical PWT in mice treated with GeXIVA[1,2] microneedle patches every three days (treatment initiated on Day 9). Groups: Control (healthy mice); Sham (sham surgery: skin and muscle incision without nerve damage); CCI (CCI model mice without treatment); CCI+MN (CCI model mice treated with GeXIVA[1,2] microneedle patches), * represents *p* < 0.05, n = 8.

We further evaluated the therapeutic efficacy of GeXIVA[1,2] microneedle patches in a CCI model of neuropathic pain. Figure 7A illustrates the experimental timeline. Figure 7B shows a progressive decrease in PWT of surgical groups (i.e., sham, CCI, and CCI + MN groups) that reached its minimum by postoperative Day 7 in CCI and CCI + MN groups. We applied microneedle patch treatment on Day 9. After treatment, mechanical allodynia was rapidly relieved, with the PWT increasing to 0.8369 ± 0.1383 g after 2 h of administration. Throughout the treatment period, the microneedle-treated group maintained PWT levels (0.7061 ± 0.1078 g), demonstrating significant improvement compared to untreated CCI mice (0.2170 ± 0.1215 g, *p* < 0.05). These findings confirm that GeXIVA[1,2] microneedle patches effectively reverse CCI-induced mechanical allodynia.

### 2.6. The Effect of GeXIVA[1,2] Microneedle Patch on Motor Function Recovery

**Figure 8:**
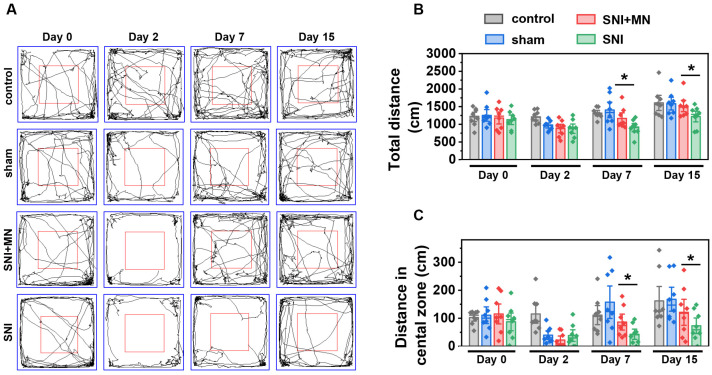
Therapeutic effect of GeXIVA[1,2] microneedle patches on locomotor ability in SNI mice. (**A**) Representative trajectory diagrams of experimental animals. (**B**) Total distance traveled. (**C**) Distance traveled in the central zone. (* represents *p* < 0.05, n = 8).

Chronic neuropathic pain is associated with impaired motor function and nerve injury-induced mobility deficits, both of which significantly reduce the quality of life and lead to long-term disability in patients [31]. We further investigated the therapeutic efficacy of GeXIVA[1,2] microneedle patches in alleviating neuropathic pain-associated locomotor dysfunction using an open field test, which can assess motor function recovery through automated video tracking and computer-assisted analysis (Figure 8). We first evaluated the therapeutic effects of GeXIVA[1,2] microneedle patches on impaired locomotor performance in SNI model mice. Figure 8A presents a representative trajectory diagram of the experimental animals, and we measured the total movement distance as well as the movement distance in the central zone for each group. As shown in Figure 8B, the total distances of the four groups were comparable before surgery (Day 0), with no statistically significant differences. However, the total distances for all groups except the control group were significantly reduced after surgery (Day 2) due to surgical injury. Following treatment with GeXIVA[1,2] microneedle patches, the locomotor ability of SNI model mice showed partial recovery. The total distances increased to 1168 ± 292 cm (Day 7) and 1518 ± 305 cm (Day 15), which were significantly greater than those of untreated SNI model mice (907 ± 220 cm and 1126 ± 267 cm, respectively). These results suggest that GeXIVA[1,2] microneedle patches can partially alleviate the impairment of autonomous locomotor ability caused by SNI.

Interestingly, analysis of the mice’s trajectories during the open field test revealed that the differences in total movement distance were primarily attributable to differences in the distance traveled in the central zone, which correlates with exploratory behavior. Exploratory ability can be influenced by both locomotor impairment and neuropathic pain. We measured the distances traveled in the central zone for all four groups. Before surgery (Day 0), the distances in the central zone were similar across groups, with no significant differences. After surgery (Day 2), the distances in the central zone were significantly reduced for three surgical groups, consistent with the trend observed for total movement distances. This reduction in central zone distance indicated that the surgery impaired the mice’s ability to explore freely. Treatment with GeXIVA[1,2] microneedle patches partially restored exploratory ability in SNI model mice, with central zone distances reaching 86.50 ± 53.55 cm (Day 7) and 121.37 ± 87.80 cm (Day 15), compared to 43.15 ± 34.42 cm (Day 7) and 73.39 ± 52.46 cm (Day 15) in untreated SNI model mice. These results suggest that GeXIVA[1,2] microneedle patches can partially mitigate the impairment of free exploration ability induced by SNI (Figure 8C).

**Figure 9:**
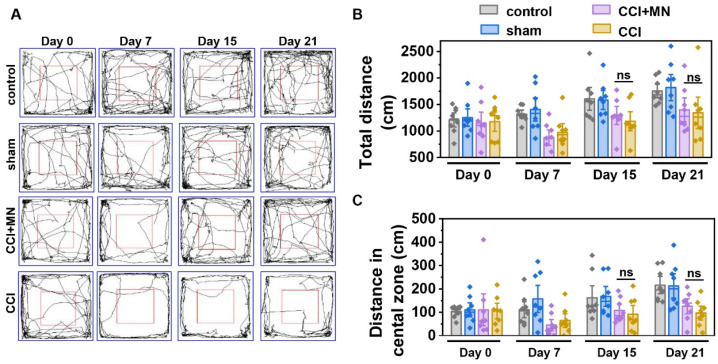
Therapeutic effect of GeXIVA[1,2] microneedle patches on locomotor ability in CCI mice. (**A**) Representative trajectory diagrams of experimental animals. (**B**) Total distance traveled. (**C**) Distance traveled in the central zone. ns represents no statistical difference (*p* > 0.05, n = 8).

We also examined open field behavior in CCI model mice, including total movement distance and distance traveled in the central zone (Figure 9). Figure 9A shows representative trajectories of the experimental animals. Figure 9B,C are the total locomotor distance and central zone distance for the four groups. Before surgery (Day 0), the distances were comparable across groups, with no significant differences. After establishing the CCI model (Day 7), the total distance and central zone distance were significantly reduced in the three surgical groups compared to the control groups, confirming that CCI and sham-operated surgery impaired both locomotor and exploratory abilities. Unlike the SNI model, CCI model mice treated with GeXIVA[1,2] microneedle patches did not show significant improvements in total distance or central zone distance on Days 15 and 21 compared to untreated CCI model mice, indicating no significant alleviation of locomotor or exploratory dysfunction by Day 21 (12 days of treatment). Histological analysis of tissue sections from the nerve injury site revealed pronounced inflammation and muscle atrophy in CCI model mice at Day 21, whereas minimal inflammation and muscle abnormalities were observed in SNI model mice (Figure S4).

Locomotor dysfunction in mice is associated with nerve injury-induced inflammation and muscle atrophy, and the CCI model exhibited more severe and persistent inflammation and muscle atrophy compared to the SNI model during treatment. Although GeXIVA[1,2] microneedle patches administered every three days significantly alleviated mechanical pain sensitivity in CCI model mice, they were insufficient to treat locomotor dysfunction caused by CCI nerve injury. This suggests that increasing the dosage of GeXIVA[1,2] or extending the treatment duration may be necessary [32]. In this study, we explored the therapeutic potential of GeXIVA[1,2] microneedle patches for alleviating locomotor dysfunction resulting from SNI and CCI. However, the quantitative relationship between dosage and effectiveness remains unclear, necessitating further in-depth studies.

## 3. Discussion

The key innovation of this study is the development of a GeXIVA[1,2] hydrogel microneedle patch for chronic neuropathic pain management. Currently, conotoxins are typically administered via intrathecal or intramuscular injections, which are invasive, technically complex, prone to infections, and poorly tolerated by patients. To address these limitations, we loaded GeXIVA[1,2] into a hydrogel microneedle patch, leveraging the advantages of microneedle technology to enable minimally invasive, painless, and patient-friendly transdermal delivery of macromolecules. This approach establishes a novel administration route for GeXIVA[1,2]. The microneedles penetrate the skin and remain embedded, where the hydrogel undergoes swelling and degradation to facilitate the slow and sustained release of GeXIVA[1,2]. This sustained-release αO-conotoxin GeXIVA[1,2] hydrogel microneedle patch can broaden the potential applications of GeXIVA[1,2] and support its preclinical development.

Our group has demonstrated the therapeutic efficacy of GeXIVA[1,2] in chronic neuropathic pain, with previous research results showing that daily intramuscular injections of GeXIVA[1,2] provide a stable analgesic effect for CCI-induced neuropathic pain [15]. However, GeXIVA[1,2] faces challenges such as rapid clearance and poor stability, especially under alkaline and thermal conditions [33]. For instance, the concentration of GeXIVA[1,2] decreased to less than 15% after 1 h of incubation with serum and became undetectable after 4 h [33]. This instability is primarily due to the rapid disruption of the disulfide bond framework in GeXIVA[1,2] by glutathione, human serum albumin, and other disulfide bond-interfering agents, leading to a short functional duration. Consequently, the analgesic effect of a single injection of GeXIVA[1,2] in previous studies lasted only 6 h, significantly limiting its utility for chronic neuropathic pain management. In this study, we encapsulated GeXIVA[1,2] in a PVA–sucrose composite hydrogel, which acts as a physical barrier, reducing GeXIVA[1,2]’s rapid degradation, enhancing its stability. Its biological activity remained for at least 3 days after a single administration. This GeXIVA[1,2] hydrogel microneedle patch reduces the frequency of administration to once every three days, minimizes blood concentration fluctuations, and extends the duration of effective analgesia. The therapeutic efficacy of the GeXIVA[1,2] microneedle patch is comparable to that of daily intramuscular injections of an equivalent dose of GeXIVA[1,2] as demonstrated in our previous research [14,15]. Thus, the GeXIVA[1,2] microneedle patch developed in this study not only prolongs the effective duration of GeXIVA[1,2] but also reduces the frequency of administration through a more convenient and patient-friendly delivery method.

In this study, we developed an extremely gentle method for preparing GeXIVA[1,2] microneedle patches to ensure that GeXIVA[1,2] retains its bioactivity during the fabrication process. The preparation of GeXIVA[1,2] hydrogel microneedle patches involves three key steps: (1) Mixing GeXIVA[1,2] with a PVA–sucrose hydrogel precursor solution, (2) cross-linking the GeXIVA[1,2]-containing hydrogel precursor solution, and (3) preparing the microneedle patch backboard layer. First, during the mixing process, we carefully controlled the stirring speed. Additionally, the inherent viscosity of the PVA–sucrose solution protects the peptides from shear force-induced damage, ensuring the stability of GeXIVA[1,2]. Second, the GeXIVA[1,2]-containing hydrogel was cross-linked using a freezing–thawing method. Specifically, the hydrogel precursor solution was frozen at −20 °C for 4 h to solidify and then thawed at 4 °C for 12 h. This freezing–thawing cycle was repeated twice. During this process, ice crystals formed at sites of concentrated hydrogen bonds, cross-linking the hydrogel precursor solution into a gel state. These ice crystals remain stable at body temperatures. Due to the abundance of hydrogen bonds in PVA and sucrose, stable cross-linking was achieved in just two cycles. Unlike chemical cross-linking methods, this physical approach avoids the use of chemical reagents, thereby preserving the peptide’s bioactivity. In our previous study, GeXIVA[1,2] retained over 99% bioactivity after six freezing–thawing cycles under −20 °C/40 °C conditions [33]. Thus, the cross-linking process does not compromise the bioactivity of GeXIVA[1,2]. Finally, for the preparation of the microneedle patch backboard, we used NOA63, which requires ultraviolet (UV) light cross-linking for 1 min (UV power = 200 W/m^2^). Our prior research has demonstrated that GeXIVA[1,2] remains stable under short-term, low-dose UV irradiation. Specifically, GeXIVA[1,2] retained over 98% bioactivity [33] after being exposed to UV light at 200 W/m^2^ for 2 days—far exceeding the irradiation time used in this study (1 min). Therefore, none of the preparation steps in this study adversely affected the bioactivity of GeXIVA[1,2]. To validate the analgesic efficacy of GeXIVA[1,2] in hydrogel microneedle patches, we conducted experiments using SNI and CCI model mice. The observed significant analgesic effects in these neuropathic pain models further confirm the success of our approach.

Currently, the invasive and complex administration routes of conotoxins limit their application to the treatment of severe diseases where intervention is essential, such as severe pain, cancer, and central nervous system degenerative diseases. However, beyond disease treatment, various types of conotoxins hold significant potential for applications in healthy individuals [34]. For example, conotoxins that block the sodium channel (Naᵥ1.4), such as μ-conotoxin PIIIA [35], play a crucial role in regulating muscle contraction. By modulating neuromuscular signaling, they can influence the tension of facial muscles, thereby reducing the appearance of dynamic wrinkles (e.g., expression lines) and achieving anti-wrinkle effects on the skin. Similarly, conotoxins that target human Naᵥ1.7, Naᵥ1.8, or α7 nAChR (such as μ-Conotoxin KIIIA [36], μ-Conotoxin MfVIA [37], [Q1G, Delta R14]LvIB [38]) can reduce skin inflammation, improving conditions like acne and eczema, enhancing overall skin health. Conotoxin-loaded microneedle patches demonstrate potential as an alternative delivery method, with advantages including convenience, minimal invasiveness, and improved patient compliance. Combined with the well-researched safety of conotoxins, these conotoxin-loaded microneedle patches are poised to gain popularity among healthy individuals for non-therapeutic applications, particularly in the field of cosmetics in the near future.

## 4. Materials and Methods

### 4.1. Materials

The PDMS kit was purchased from Dow Corning (Midland, MI, USA). Designed copper microneedle molds were obtained from Guangzhou LAKEY Micromachining Center (Guangzhou, China). PVA (Mw 27 000) and sucrose were purchased from Macklin Biochemical Co., Ltd. (Shanghai, China). HA, methylene blue, and PBS were obtained from Aladdin Co., Ltd. (Beijing, China). Paraformaldehyde, acetonitrile, dextran, and trifluoroacetic acid (TFA) were purchased from Thermo Fisher Scientific (Pittsburgh, PA, USA). NOA63 was purchased from Norland Products Incorporated (Cranbury, NJ, USA). Mice were supplied by Guangxi Medical University Laboratory Animal Center (Nanning, China). GeXIVA[1,2] was synthesized and modified by our research group, and the detailed protocols can be found in our previous publications [11].

### 4.2. Fabrication of GeXIVA[1,2] Hydrogel Microneedle Patches

**Figure 10:**
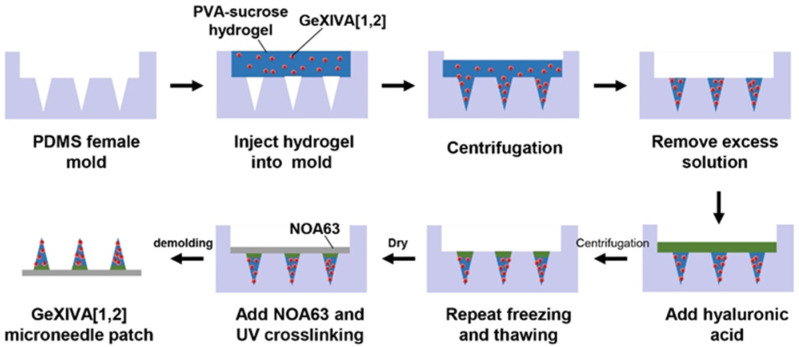
Schematic representation of the fabrication steps of the GeXIVA[1,2] hydrogel microneedle patches.

PDMS mixed with 10% (*w*/*w*) curing agent was cast into a copper microneedle mold, cured at 60 °C for 1 h, and then demolded to form a PDMS female mold. PVA and sucrose were dissolved in hot water at 90 °C in a mass ratio of 4:2 to 4:6 to form a 56% PVA–sucrose solution. After the temperature was lowered to the room temperature, GeXIVA[1,2] was added and mixed well to obtain a hydrogel solution at a concentration of 80 μM; 500 μL of the mixed hydrogel solution was added into a PDMS female mold, centrifuged for 10 min at 3200 rpm, and then kept under vacuum for 5 min. Subsequently, the excess solution outside the mold’s micropores was removed for reuse, leaving approximately 25 μL of the solution within the mold (2 nmol of GeXIVA[1,2] per microneedle patch). After the microneedle tips were completely dried, 50 μL of 2.5% HA solution was added into the microneedle mold and centrifuged at 3000 rpm for 5 min. The microneedle mold containing the hydrogel solution was frozen at −20° C for 4 h, thawed at 4 °C for 12 h, with this cycle repeated twice to cross-link the PVA–sucrose hydrogel, and then dried in a vacuum drying oven overnight. Finally, 250 μL of NOA63 solution was added on top of the mold and cross-linked by UV for 1 min to form the microneedle patch backboard. The GeXIVA[1,2] microneedle patch was prepared after careful demolding and was kept in a vacuum oven at 4 °C (Figure 10).

### 4.3. Exploration of Optimal Ratio of PVA–Sucrose Hydrogel

Hydrogel microneedle patches were prepared using PVA:sucrose ratios of 4:2 to 4:6 (*w*/*w*) and their morphology was characterized using optical microscopy. The integrity of hydrogel microneedle patches was assessed using the formula: N/100 × 100%, where N represents the number of intact microneedles in a patch, and ten different microneedle patches per group were evaluated. PVA–sucrose hydrogel blocks were embedded subcutaneously in isolated mice tissues to evaluate their swelling ability. The swelling ratio was calculated according to the formula: (M − M_0_)/M_0_, while M_0_ and M are the mass of PVA–sucrose hydrogel before and after embedded in mice tissues for 0 to 72 h, (n = 3).

### 4.4. Characterizations of GeXIVA[1,2] Microneedle Patch

Methylene blue was used instead of conotoxin GeXIVA[1,2] in the preparation of microneedle patches to visualize drug loading and the morphological changes of the microneedles [24]. Microneedle patches were photographed with a camera (Nikon D90, Nikon Corporation, Tokyo, Japan), optical microscopy (Eclipse LV100NDA LED, Nikon Corporation, Tokyo, Japan), and tabletop scanning electron microscope (SEM) (Hitachi TM 4000 Plus, Hitachi High-Technologies Corporation, Tokyo, Japan). The height and diameter of the microneedles were measured using ImageJ (V1.8.0) through photographs taken with an optical microscope. The skin penetration ability of hydrogel microneedle patches was evaluated by inserting them into depilated mice’s back skin and microholes formed as well as mode drugs released in the mice skin were recorded by a camera. Then the mice were sacrificed and skin tissue was fixed in 4% paraformaldehyde and stained by H&E for the histological analysis. The insertion rate was calculated as the number of microneedles successfully inserted into the skin divided by the total number of microneedles in a patch. The separation rate was calculated as the number of microneedles remaining under the skin divided by the total number of microneedles in a patch. Images of the microneedle patches before and after insertion into the skin were obtained by camera and microscope.

### 4.5. In Vitro Release Study of GeXIVA[1,2] from Hydrogel Microneedle Patches

Two nmol GeXIVA[1,2]-loaded microneedle patches without an HA layer were prepared for detection of GeXIVA[1,2] release in vitro. Microneedle patches were placed in 10 mL PBS under stirrer (100 rpm) at 37 °C. At each designated time point (from 1 min to 48 h), 200 μL of the supernatants were collected by centrifugation at 4000 rpm for 10 min and replaced with an equal volume of fresh PBS. The concentration of GeXIVA[1,2] in the supernatant was determined by the RP-HPLC system according to previous studies [11,39]. The RP-HPLC column was SymmetryShield RP18 5 μm × 4.6 mm × 150 mm, and the mobile phase consisted of 90% acetonitrile and 0.05% TFA; 80 μL samples were injected into the RP-HPLC system and eluted with a flow rate of 1.0 mL/min at 40 °C. The detection wavelength was set at 214 nm.

### 4.6. Drug Release In Vivo

To visualize drug release in vivo, dextran (Mw = 3000 Da), which had similar molecular weight as the GeXIVA[1,2], modified with F8BT fluorescent groups was used as model drugs to fabricate a hydrogel microneedle patch. These microneedle patches were applied in the depilated back skin of mice and captured images using an In Vivo Animal Imaging System (Revvity IVIS Lumina LT Series III) with a 500 nm excitation light and a bandpass filter from 570 to 650 nm. Then fluorescence intensity was analyzed.

### 4.7. Mechanical Allodynia Testing

Male C57BL/6J mice (∼6 weeks) with an average weigh of 20 ± 2 g were purchased from Guangxi Medical University. All of the animal studies were performed in compliance with guidelines set by the Institutional Animal Care Committee of Guangxi University (NO. GXU-2024-207). Mechanical allodynia was assessed by measuring the mechanical PWT using the Von Frey test method after the mice were purchased and acclimated to the environment for one week. Plastic cages with a wire mesh base that were suspended above a table were used to measure mechanical PWT. Prior to testing, the mice were placed in cages for 30 min to allow them to calm down. A set of Von Frey filaments was used to vertically stimulate the plantar surface of the left hind limb and pain-related behaviors (paw withdrawal or licking the hind limb) were observed. If the mice show a pain response, the filaments were replaced with the lower grammage one, and vice versa. Following the crossing of the measurements, four subsequent tests were performed. The resulting six data points were analyzed using the up–down method to determine the 50% mechanical PWT [40]. Daily mechanical PWT measurements were performed until the values stabilized (approximately one week). Mice unable to complete the paw withdrawal reflex were excluded. The remaining mice were randomly divided into two groups for SNI and CCI treatment studies. Each group was then divided into four subgroups (n = 8): (1) control, (2) sham-operation, (3) SNI (or CCI) surgery, and (4) SNI (or CCI) surgery and microneedle treatment.

### 4.8. Animal Chronic Neuropathic Pain Models

After the mechanical PWT values were stabilized, SNI, CCI or sham surgical procedures were performed. The SNI model was produced according to Richner’s study [41]. Briefly, mice were deeply anesthetized by intraperitoneal injection of sodium pentobarbital (80 mg/kg). The skin was opened, and the muscle layer was separated using blunt dissection to visualize the left sciatic nerve. The area where the sural nerve branches from the sciatic nerve was identified. Two of the three branches of the sciatic nerve (the tibial nerve and the common peroneal nerve) were ligated using 4-0 chromic gut ligatures with tight surgical knots, and were transected distal to the ligature using surgical forceps. The smallest of the three branches (sural nerve) was left intact. Then the muscle and skin were sutured layer by layer, and penicillin sodium powder was applied to prevent infection. The CCI model was produced according to Bennett and Xie’s study [42]. Briefly, mice were deeply anesthetized by intraperitoneal injection of sodium pentobarbital (80 mg/kg). Then the left-side sciatic nerve was exposed at the mid-thigh level, and was loosely tied using four chromic gut ligatures (4-0) at 1 mm intervals. The ligation strength was sufficient to constrict the nerve but not to interfere with blood transport in the nerve periphery. Then the muscle and skin were sutured layer by layer, and penicillin sodium powder was applied to prevent infection. The sham-operation group involved opening the skin and separating the muscles on the left side, exposing the sciatic nerve but not severing or ligating the nerve. Then similarly suture muscles and skin layer by layer and penicillin sodium powder was applied to prevent infection. The mechanical PWT was continuously monitored.

### 4.9. Administration of GeXIVA[1,2] Microneedle Patches

Administration of microneedle patches began on the third day (SNI group) and ninth day (CCI group) after the surgical procedure and was repeated every three days. For microneedle patch application, the skin at the surgical site was pinched up and then the microneedle patch was pressed with the thumb to pierce into the skin, held for 1 min to peel off the backboard of the microneedle patch. Mechanical allodynia was measured two hours after administrating GeXIVA[1,2] microneedle patches and continuously measured for 20 days postoperatively in the SNI group or for 23 days in the CCI group.

### 4.10. Open Field Test

The open field test was performed at postoperative days 0, 2, 7, and 15 in the SNI group and at days 0, 7, 15, and 21 in the CCI group. Mice were acclimated to the testing environment for 30 min before the open field test. The open field test apparatus consisted of a square chamber (40 × 40 × 40 cm). Each mouse was placed in the center and allowed to freely explore for 5 min. Behaviors of mice, including total movement distance and distance in the central zone were recorded and analyzed using SMART 3.0 software (Panlab Harvard Apparatus, Barcelona, Spain). Upon completion of the animal behavior experiments, the mice were sacrificed. The sciatic nerve tissues were harvested, fixed with 4% paraformaldehyde, embedded in paraffin, and prepared into tissue sections. Histological analysis was conducted after H&E staining.

### 4.11. Statistical Analysis

All quantitative results in vitro were obtained from at least 3 samples, while animal studies consisted of 8 samples. Data were expressed as the mean ± standard error. A two-tailed paired Student’s t-test was used to compare the differences. Difference with *p* < 0.05 was considered to be statistically significant.

## Data Availability

The data presented in this study are available on request from the corresponding author.

## Supplementary Materials

The following supporting information can be downloaded at: https://www.mdpi.com/article/10.3390/md23040161/s1, Figure S1: RP-HPLC and electrospray-ionization mass spectroscopy (ESI-MS) profiles of synthesized GeXIVA[1,2]; Figure S2: In vitro release of the model drug (methylene blue)-loaded microneedle patches; Figure S3: Skin appearance of mouse before and after microneedle patch application; Figure S4: Images of H&E-stained sections of sciatic nerve tissue.; Video S1: A hydrogel microneedle patch with detachable needle tips and backing board.

## Abbreviations

The following abbreviations are used in this manuscript:

nAChR	nicotinic acetylcholine receptor
PVA	polyvinyl alcohol
NOA63	Norland optical adhesive 63
HA	hyaluronic acid
RP-HPLC	reverse-phase high-performance liquid chromatography
ESI-MS	electrospray-ionization mass spectroscopy
PBS	phosphate buffer saline
SNI	spared nerve injury
CCI	chronic constriction injury
PWT	paw withdrawal threshold
UV	ultraviolet
PDMS	polydimethylsiloxane
TFA	trifluoroacetic acid
SEM	scanning electron microscope
H&E	hematoxylin–eosin
