# Supplementary material for: Sustained Release of αO-Conotoxin GeXIVA[1,2] via Hydrogel Microneedle Patch for Chronic Neuropathic Pain Management
Source: Mar Drugs. 2025 Apr 7;23(4):161. doi: 10.3390/md23040161 (PMC12028434; doi:10.3390/md23040161)
Supplement: Supplementary file 1 [file marinedrugs-23-00161-s001.zip › Supplementary material.docx]

**Supplementary Material**

**Sustained release** **of αO-conotoxin GeXIVA[1,2] via hydrogel microneedle patch for chronic neuropathic pain management**

Rongyan He^1^, Mingjuan Li^1^, Weitao Li^1^, Wenqi Li^1^, Shuting Xiao^1^, Qiuyu Cao^1^, Huanbai Wang^1^, Dongting Zhangsun^1, 2^, Sulan Luo^1, 2^*

1 Guangxi Key Laboratory of Special Biomedicine, School of Medicine, Guangxi University, Nanning, 530004, China.

2 Key Laboratory of Tropical Biological Resources of Ministry of Education, Hainan University, Haikou 570228, China.

* Corresponding authors

E-mail addresses: sulan2021@gxu.edu.cn


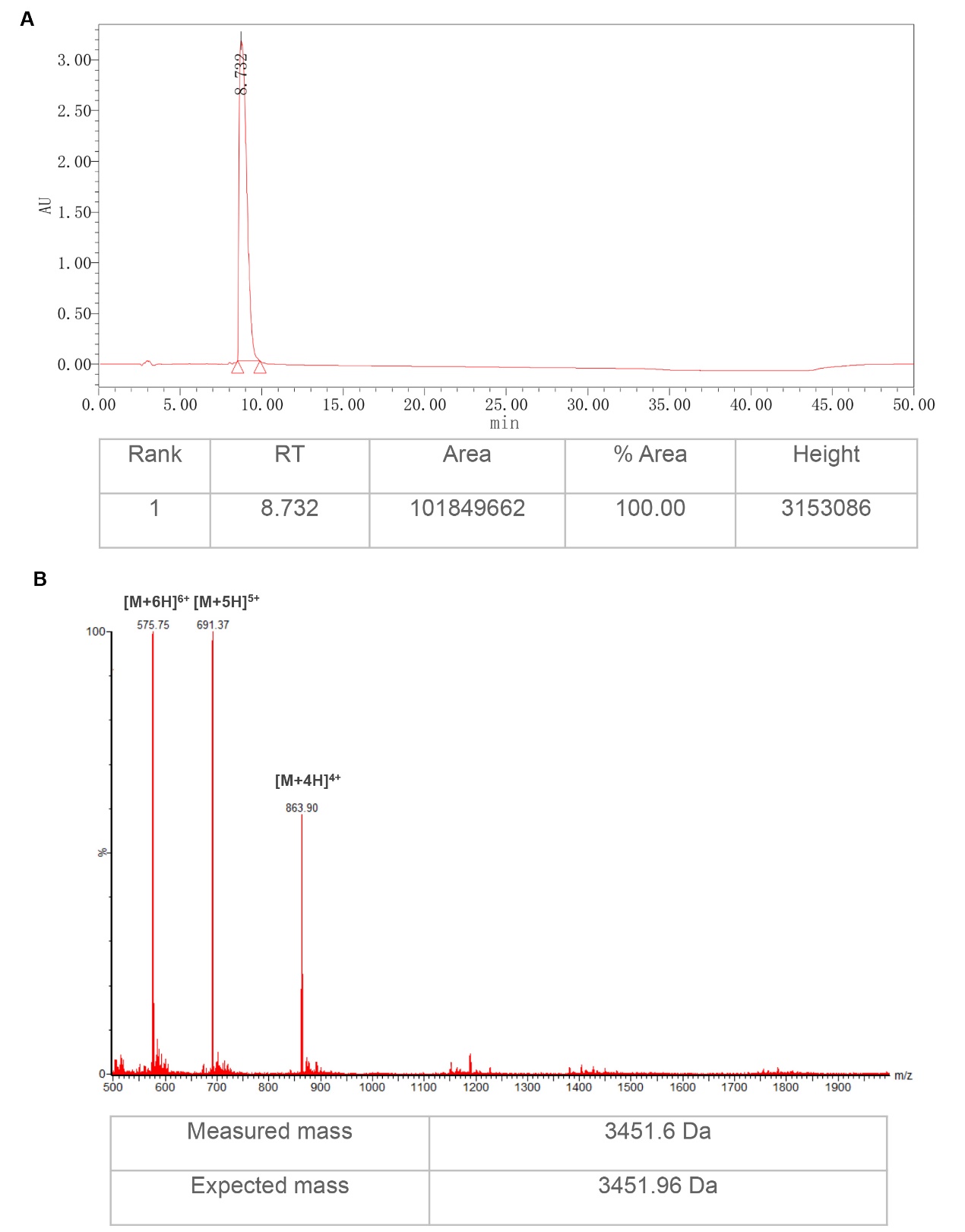


**Figure S1**. RP-HPLC (A) and electrospray-ionization mass spectroscopy (ESI-MS) (B) profiles of synthesized GeXIVA[1,2].


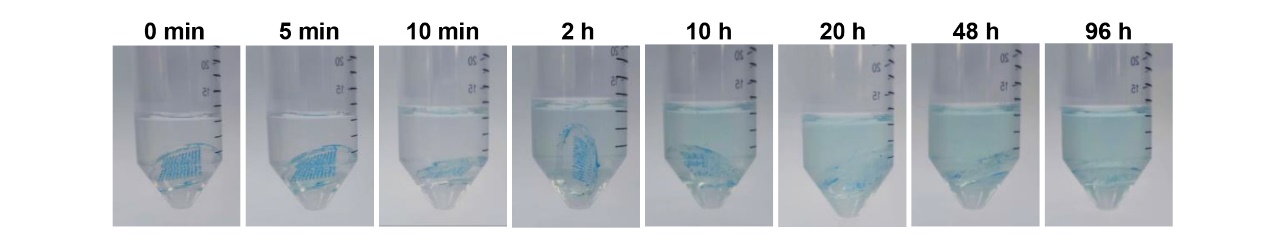


**Figure S2**. ***In vitro* release of the model drug (methylene blue)-loaded microneedle patches.**


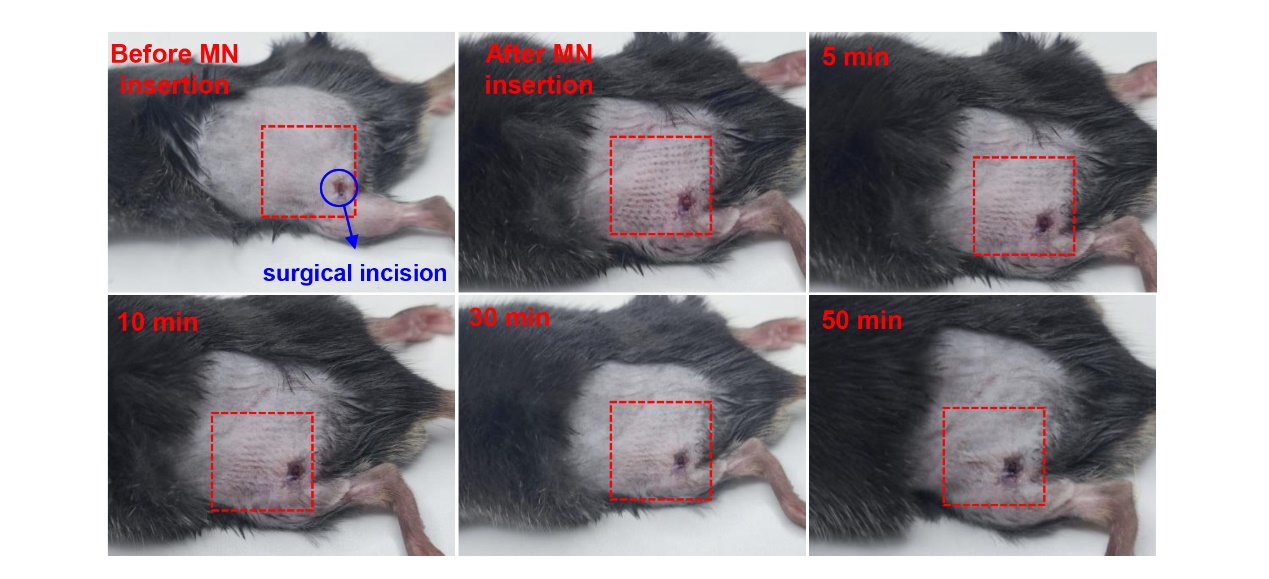


**Figure S3**. **Skin appearance of mouse before and after microneedle patch application.** A localized micropore array is visible at the application site immediately after patch removal, which fully resolves within 50 min, restoring the skin to its original state. The red box marks the microneedle application site, while the wound circled in blue is from SNI modeling, not caused by the microneedle patch.


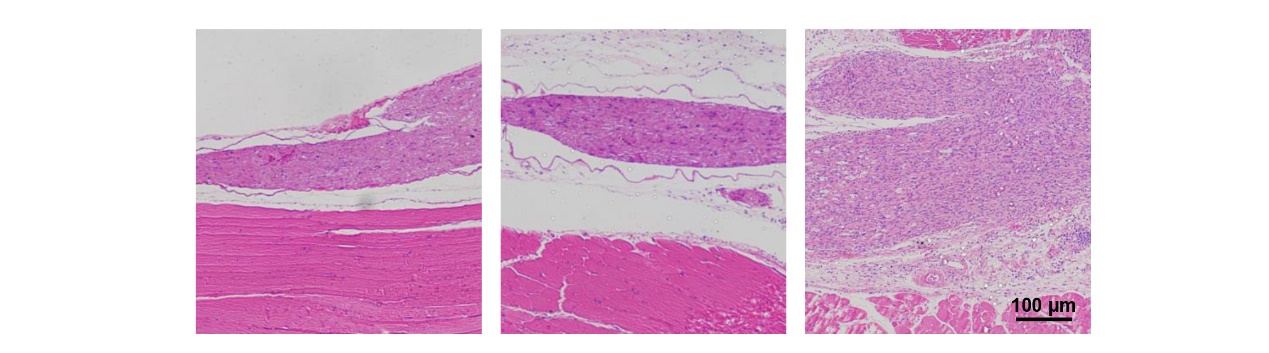


**Figure S4**. **Images of H&E-stained sections of sciatic nerve tissue.** From left to right: control mice, SNI model mouse treated with GeXIVA[1,2] microneedle patch, and CCI model mouse treated with GeXIVA[1,2] microneedle patch for 12 days.
